# Design of Enzyme Loaded W/O Emulsions by Direct Membrane Emulsification for CO_2_ Capture

**DOI:** 10.3390/membranes12080797

**Published:** 2022-08-18

**Authors:** Suchintan Mondal, Bhavna Alke, Aline Machado de Castro, Paloma Ortiz-Albo, Usman Taqui Syed, João G. Crespo, Carla Brazinha

**Affiliations:** 1 LAQV/Requimte, Department of Chemistry, NOVA School of Science and Technology, FCT NOVA, Universidade NOVA de Lisboa, 2829-516 Caparica, Portugal; 2 Research and Development Center, PETROBRAS, Av. Horácio Macedo, 950. Ilha do Fundão, Rio de Janeiro 21941-915, Brazil

**Keywords:** membrane emulsification, water-in-oil emulsions, CO_2_ recovery from biogas, carbonic anhydrase, emulsion-based supported liquid membrane

## Abstract

Membrane-based gas separation is a promising unit operation in a low-carbon economy due to its simplicity, ease of operation, reduced energy consumption and portability. A methodology is proposed to immobilise enzymes in stable water-in-oil (W/O) emulsions produced by direct membrane emulsification systems and thereafter impregnated them in the pores of a membrane producing emulsion-based supported liquid membranes. The selected case-study was for biogas (CO_2_ and CH_4_) purification. Upon initial CO_2_ sorption studies, corn oil was chosen as a low-cost and non-toxic bulk phase (oil phase). The emulsions were prepared with Nadir^®^ UP150 P flat-sheet polymeric membranes. The optimised emulsions consisted of 2% Tween 80 (*w*/*w*) in corn oil as the continuous phase and 0.5 g.L^−1^ carbonic anhydrase enzyme with 5% PEG 300 (*w*/*w*) in aqueous solution as the dispersed phase. These emulsions were impregnated onto a porous hydrophobic PVDF membrane to prepare a supported liquid membrane for gas separation. Lastly, gas permeability studies indicated that the permeability of CO_2_ increased by ~15% and that of CH_4_ decreased by ~60% when compared to the membrane without carbonic anhydrase. Thus, a proof-of-concept for enhancement of CO_2_ capture using emulsion-based supported liquid membrane was established.

## 1. Introduction

Global warming, the direst threat to the planet, has been a highly debated topic all over the world. Carbon dioxide (CO_2_) is considered to be the major contributor to global warming mainly due to its increased production during the combustion of fuels for the generation of power [1]. To avoid the catastrophic climatic changes brought about by the emission of CO_2_ into the atmosphere, research in the field of carbon capture and storage has been progressing rapidly. Another application of CO_2_ capture is encountered in the purification of biogas, a carbon neutral alternative renewable energy resource [2]. Biogas is mainly constituted by CH_4_ and CO_2_ along with several contaminants such as ammonia (NH_3_), carbon monoxide (CO), water vapour, methyl siloxanes, hydrogen sulfide (H_2_S), nitrogen (N_2_), oxygen (O_2_), halogenated volatile organic compounds (VOCs) and hydrocarbons. It is crucial to purify biogas by removing the contaminants (mainly CO_2_ and H_2_S) in order to increase the calorific value and upgrade it to be used as a cleaner fuel [3].

Pre-combustion, oxyfuel combustion and post-combustion are the three major technologies adapted for CO_2_ capture and storage and CO_2_ conversion and utilisation. Post-combustion carbon capture technologies are the most common techniques used for CO_2_ capture. These include absorption, adsorption, membrane-based separation and enzyme-based methods [4,5]. Amine-based carbon capture (e.g., with functional amines such as monoethanolamine-MEA, N-methyldiethanolamine-MDEA) [6] is the most widely used technique of absorption [4,7]. Membrane-based separation includes the utilisation of membrane contactors, high temperature and permselective polymers and blend membrane materials, whereas enzyme-based methods involve enzyme catalytic hydrolysis and carbonic anhydrase [4,8,9,10].

When using enzymatic systems, the success of CO_2_ capture and storage and CO_2_ conversion are highly dependent on the ability to convert (transfer) CO_2_ to an aqueous phase from the gas stream. Carbonic anhydrase, which has a hydroxide-bound Zn^2+^ in its active site, catalyses the inter-conversion between carbon dioxide and bicarbonate promoting the above mentioned requirement [11]. Carbonic anhydrase has been known to promote CO_2_ absorption and desorption, leading to an improvement in the reaction kinetics and further reduction in the cost of energy required to release the absorbed CO_2_ in solvent-based CO_2_ capture [12]. Though the utilisation of carbonic anhydrase has its benefits, factors such as the presence of impurities, high salt concentration during absorption and the poor stability of the enzyme at alkaline pH and high temperature, restricts its use at an industrial level for CO_2_ capture. Hence, the study of processes using carbonic anhydrase for CO_2_ capture is still a highly researched topic [11].

Membranes are permselective barriers between two phases [13,14]. Liquid membranes (LMs), as the name suggests, are membranes usually comprising at least one liquid component in it [15]. The advancement in liquid membrane technology is credited to Li et al., who patented emulsion liquid membranes (ELMs) for applications in hydrocarbon separation and desalination at an industrial scale in the year 1968 [16,17]. Bulk liquid membranes (BLMs), supported liquid membranes (SLMs) and emulsion liquid membranes are the three main categories of liquid membranes [15]. Among these, ELMs are currently used extensively in extraction and separation-based applications [15]. Basically, ELMs are systems in the form of double emulsions (water-in-oil emulsion dispersed in an external aqueous phase or oil-in-water emulsion dispersed in an outer organic phase) [18]. However, the physical instability of the ELMs and the de-emulsification processes required is still a major disadvantage from practical industrial perspective [18]. Additionally, formation of double emulsions in comparison to simple emulsions involves 2 cycles of emulsification, which makes it an energy-intensive process. The combination of the principles of SLMs with tailor-made emulsions as a functional solvent sets the premise of the present study.

Emulsions have been formed by both high-energy methods and low-energy methods. While high-energy methods include those methods which use mechanical devices that are highly energy intensive, such as ultrasonicators, microfluidisers and high-pressure homogenisers, low-energy methods are those which form emulsions as a result of structural changes and processing conditions at the interfaces, such as by spontaneous emulsification, bubble bursting and phase inversion temperature methods [19]. Among the high-energy methods, ultrasound-assisted emulsification is preferred, as it comparatively consumes lower energy with a reduced risk of contamination by the experimental unit [20]. However, a technique that has garnered interest in recent years in the field of emulsification is membrane emulsification, a low-energy method performed at low shear stress and mild operating conditions [19]. This technique seems to be more effective in terms of productivity and energy consumption for producing emulsions with smaller sizes and controlled dispersity. Detailed comparison of membrane emulsification with traditional methods such as mechanical stirrers, homogenisers and ultrasonicators has been recently reported [19,21,22,23,24].

Liquid membranes have been used in the past for applications such as acid recovery and dye removal from aqueous solutions and extraction of metals [25,26,27,28,29]. Furthermore, the emulsions in these studies were produced by techniques involving homogenisers, high-speed mixers and ultrasonicators. The present study is an attempt to produce emulsions using the more effective membrane emulsification technique and using them to prepare emulsion-based supported liquid membranes. Additionally, this research work also explores these membranes for gas-based applications by evaluating their gas transport properties. In the present study, various commercial oils with potential CO_2_ capture ability were initially screened to be used as the continuous phase for the formation of water-in-oil emulsions by membrane emulsification. Prior to these emulsification experiments, the continuous phase, the chosen dispersed phase and the polymeric membrane to be used were characterised in terms of interfacial tension between two phases and the static contact angle of the continuous phase on the membrane surface. To evaluate the effect of carbonic anhydrase on the CO_2_ capture ability of the system, emulsions were produced both without and with the functional enzyme. Furthermore, supported liquid membranes (SLMs) were prepared by impregnation of oil onto a porous membrane support, and emulsion-based supported liquid membranes were formed by impregnation of the produced emulsion onto a porous membrane support. Finally, gas permeability measurements of CO_2_ and CH_4_ across the synthesised SLMs were performed.

## 2. Materials and Methods

### 2.1. Materials

The commercial oils screened were olive oil (Gallo, Alges, Portugal), sunflower oil (Fula, Alges, Portugal) and corn oil (Fula, Alges, Portugal). Surfactants used for emulsification were PEG 300 (Clariant, Muttenz, Switzerland), Span^®^ 80, Tween^®^ 80, Triton™ X-100 Detergent and SDS (≥99% (GC), dust-free pellets), which were procured from Sigma Aldrich, Portugal (made in Lyon, France). Potassium carbonate (>99.5% pure, Scharlab S.L., Sentmenat, Spain) was used to prepare a solution to adjust the pH to 11. Carbonic anhydrase from bovine erythrocytes (≥95% (SDS-PAGE), specific activity ≥3500 W-A units/mg protein, lyophilised powder) was acquired from Sigma Aldrich, Portugal (made in St. Louis, MI, USA). The gases used, carbon dioxide (98% purity) and methane (99% purity) were purchased from Praxair (Huelva, Spain). The commercial membranes used in this study are detailed in Table 1.

### 2.2. Schematic Representation of Experimental Workplan

**Figure 1:**
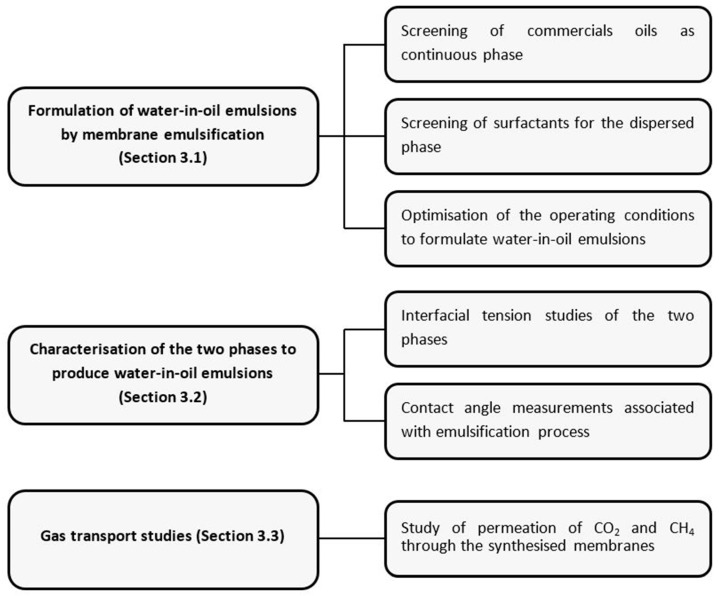
Schematic representation of experimental study.

A scheme representing the series of experiments performed in order to obtain a functionalised membrane with favourable gas transport properties is detailed in Figure 1.

### 2.3. Optimisation of the Two Phases to Produce Water-in-Oil Emulsions

#### 2.3.1. Screening of Commercials Oils as Continuous Phase

**Figure 2:**
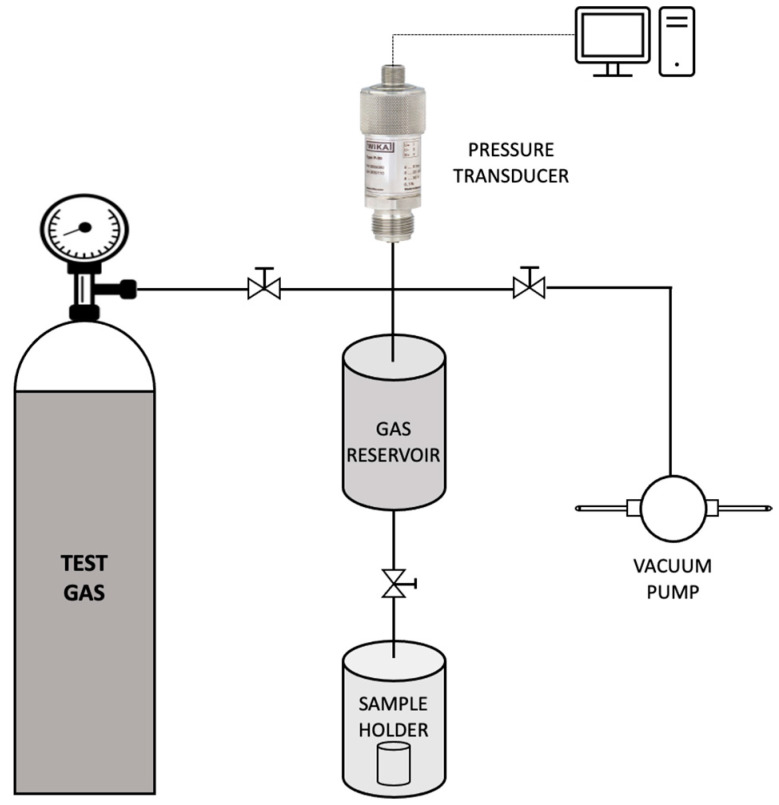
Schematic representation of the sorption unit used in the study.

Commercial oils such as olive oil, sunflower oil and corn oil were initially screened to be used as the continuous phase of the emulsion. These oils were preferred since they meet the requirement for a cheap, sustainable and functional bulk carrier. Additionally, since the aim of this study was to obtain a supported liquid membrane with high permeability and selectivity for CO_2_ capture, it was essential to choose a continuous phase containing a bulk carrier having a high affinity towards CO_2_. Thus, to check the CO_2_ uptake capacity, sorption studies with the above-mentioned commercial oils were carried out using a dual-volume sorption unit as illustrated in Figure 2. The set-up consisted of a sample holder and a storage vessel (gas reservoir). A pressure transducer connected to the gas reservoir measured the pressure in the storage vessel in real-time.

For the evaluation of sorption capacity, the sample holder (containing the sample) was initially set under vacuum conditions for about 24 h to release any gases trapped within. Subsequently, the valve to the sample holder was closed and carbon dioxide gas was supplied into the storage reservoir up to a pressure of 160.1 kPa. Once the pressure was stabilised, the valve to the sample holder was opened and the subsequent pressure decay was recorded. The amount of gas sorbed and the resulting sorption coefficient was evaluated by the Equations (1) and (2), respectively.

(1) $$n_{i,\;sample}=\frac{(P_{o}\times V_{R})-P_{t}\times \left(V_{R}+V_{S}-V_{sample}\right)}{RT}\;$$

In the Equation (1), ‘*n_i,sample_*’ [mol] is the number of moles of gas ‘*i’* sorbed onto a given sample. ‘*P_o_’* [atm] and ‘*P_t_’* [atm] are the initial pressure of gas in the storage vessel and the pressure at time ‘*t’* [s] after the gas is introduced into the sample holder, respectively. ‘*V_R_’* [m^3^] and ‘*V_S_’* [m^3^] are the volumes of the storage vessel and the sample holder, respectively. ‘*R’* [cm^3^atm·K^−1^mol^−1^] refers to the universal gas constant and ‘*T’* [K] the absolute temperature at which the experiment was performed.

The sorption coefficient ‘*S_i_’* [m^3^ (*STP*)m^3^*_sample_*atm^−1^] was evaluated as follows:

(2) $$S_{i}=\frac{V_{i,\;sample}\left(STP\right)}{V_{sample}\times P_{stabilised}}\;$$

where, ‘*V_i,sample_*‘ [m^3^] is the volume of gas ‘*i’* corresponding to ‘*n_i,sample_*’ at *STP* conditions (1 atm, 273.15 K). ‘*P_stabilised_*’ [atm] is the pressure stabilised within the dual-volume sorption unit at equilibrium conditions.

Furthermore, for the formation of emulsions, surfactants of different hydrophilic and lipophilic balance (HLB) were tested as a component with the selected commercial oil to constitute the continuous phase. Specifically, Tween 80, Span 80 and an equimolar mixture of them were tested for emulsification. 2% (*w*/*w*) was optimised as the concentration of Tween 80 in the continuous phase (data not shown).

#### 2.3.2. Selection of Dispersed Phase

The dispersed phase consisted of ultrapure MilliQ^®^ IQ water along with the carbonic anhydrase enzyme as the functional compound. Carbonic anhydrase has been reported to have high enzymatic activity at ~pH 10–11 [30]. Therefore, the pH of the dispersed phase was accordingly adjusted using potassium carbonate salt. The concentration of carbonic anhydrase used was previously optimised as 0.5 gL^−1^. Moreover, several surfactants, namely SDS, Tween 80, Triton X-100 and PEG 300, were tested to stabilise the carbonic anhydrase and facilitate the emulsion formation. The influence of the surfactants on the carbonic anhydrase was studied using fluorescence-anisotropy technique as described by Castro et al. [30] Fluorescence emission measurements were recorded using a Spex Fluorolog 3 spectrofluorometer (Horiba, Kyoto, Japan) with an excitation wavelength (λ_ex_) of 295 nm and emission wavelength (λ_em_) from 300–550 nm with excitation and emission slits of 5 nm. Datamax software was used to acquire the data.

#### 2.3.3. Characterisation of the Optimised Phases and the Membrane Used for Emulsification Studies

##### Interfacial Tension Studies

A reduced interfacial tension between the dispersed phase and the continuous phase used in a membrane emulsification study promotes the formation of emulsions [24]. Hence, the interfacial tension between the two phases was determined using a Drop Shape Analyzer (DSA 25B, Kruss GmbH, Hamburg, Germany). The fit of the pendant drop profile obtained from the software and the Laplace–Young equation was used to calculate the interfacial tension values. The measurements were carried out in triplicate at room temperature (21 ± 1 °C) and the resulting average values were considered.

##### Contact Angle Studies

During membrane emulsification studies, the dispersed phase should not wet the active layer of the membrane (where the droplet detachment occurs) to avoid coalescence on the membrane surface. Furthermore, it is imperative that the continuous phase wets the membrane surface to facilitate the detachment of the emulsions from the surface [23]. The measurement of the static contact angle of the membrane under study with the liquid phases employed further ensures the suitability of the membrane for the emulsification process. Therefore, the abovementioned Drop Shape Analyzer was used to perform the required static contact angle measurements by sessile drop method. 

### 2.4. Formulation of Water-in-Oil Emulsions by Membrane Emulsification

**Figure 3:**
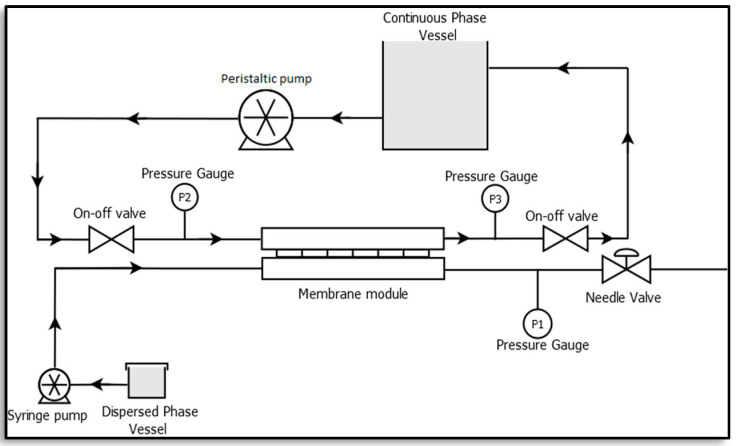
Membrane emulsification set-up [23].

A membrane emulsification unit was set-up at the lab scale as illustrated in Figure 3 [23,24]. It consists of a rectangular membrane module made of stainless steel which lodges a membrane of active area 2.9 × 10^−4^ m^2^, a syringe pump (Harvard Apparatus, PHD ULTRA 4400 I/W PROG, Cambridge, MA, USA) and a peristaltic pump (Velp Scientific, SP311, Usmate Velate, Italy). A constant volume of dispersed phase, resulting in a defined flux, was injected through the membrane into the continuous phase by the syringe pump, while the peristaltic pump was used to recirculate the continuous phase. 

The automated syringe pump was used to set the dispersed phase flow rate, ‘*Q_DP_*’ [m^3^.s^−1^] while the continuous phase cross flow velocity, ‘*ν_c_*’ [m.s^−1^] was calculated as follows [23,24]:

(3) $$\nu_{c}=\frac{4*Q_{CP}}{\pi *D_{h}^{2}}$$

where ‘*Q_CP_*’ [m^3^.s^−1^] is the flow rate of the continuous phase and ‘*D_h_*’ [m] is the hydraulic diameter of the flow channel. 

Next, 1%, 2% and 5% of dispersed phase to continuous phase volume ratios were tested for the formation of emulsions. For the emulsification process, the dispersed phase was forced through the membrane using the syringe pump while the continuous phase was recirculated using a peristaltic pump in the system. The dispersed phase passes through the membrane pores because of the applied transmembrane pressure and disperses into the continuous phase to form water-in-oil emulsions. For the optimised emulsification process, the transmembrane pressure went up to ~149.9 kPa. The emulsions formed were further characterised using an optical microscope (H550S, Nikon, Japan).

### 2.5. Preparation of Supported Liquid Membranes

**Figure 4:**
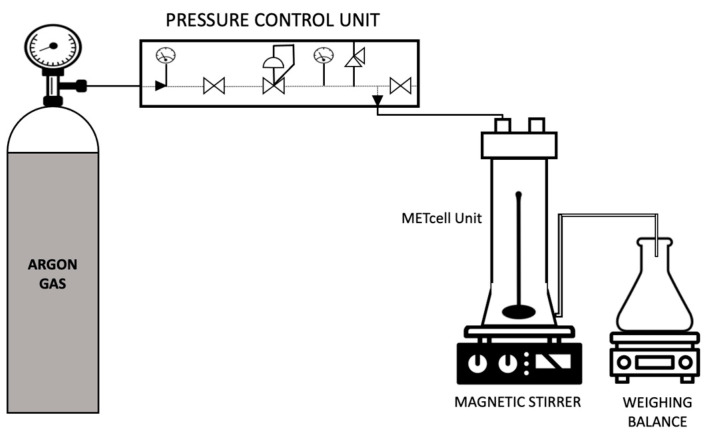
Schematic representation of the set-up for preparation of supported liquid membranes.

The liquid membranes were prepared by filling the pores of a hydrophobic PVDF Durapore^®^ membrane filter (MilliporeSigma, Burlington, MA, USA) of nominal pore size 0.22 µm with the emulsion solutions previously formed. This procedure was accomplished in a dead-end stainless steel METCell set-up as depicted in Figure 4 (Membrane Extraction Technology, London, UK). The experiments were performed at a constant controlled pressure of 395.2 kPa with a porous, stainless steel disc supporting the membrane filter. At first, the feed solution was poured over the membrane into the METcell unit. Then it was pressurised through the membrane pores by an inert gas (Argon) until 90% of the feed solution was collected in the permeate chamber. The excess solution was gently removed with a tissue paper and the membranes were subsequently air-dried in a desiccator. The membrane was weighed before and after this procedure to determine the amount of feed emulsion solution that has been incorporated into the membrane pores. 

### 2.6. Gas Transport Studies

**Figure 5:**
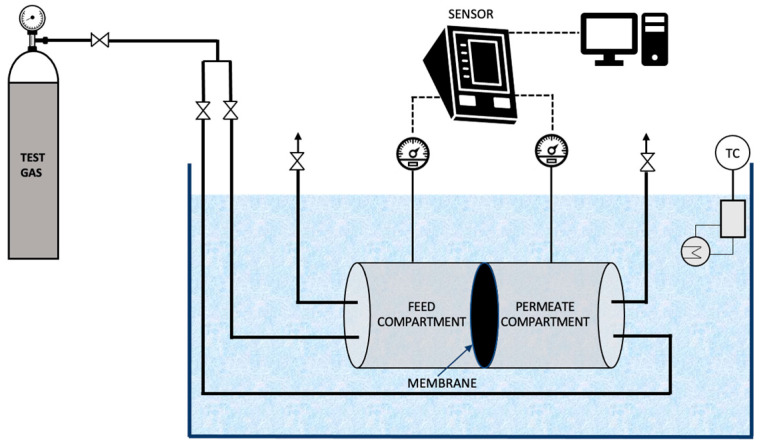
Schematic representation of the gas permeation unit used in the study.

The single gas permeability of CO_2_ and CH_4_ through the prepared membranes was measured using a gas permeation unit as described elsewhere [31]. The membrane permeation cell consists of two identical compartments, made of stainless steel, separated by the membrane to be tested as illustrated in Figure 5. The permeation cell was kept in a thermostatic water bath to maintain a constant temperature during the experiment. Initially, the gas under study (CO_2_ or CH_4_) was introduced into both the compartments until the pressure was stabilised. Subsequently, the required transmembrane pressure was established. Pressure transducers were used to measure the variation in pressure with time in both the compartments. An in-house developed software was used to monitor the pressure and acquire the data.

The permeability of a gas through the membrane was calculated [31] by Equation (4)

(4) $$\frac{1}{\beta }ln\frac{P_{feed_{0}}-P_{perm_{0}}}{P_{feed}-P_{perm}}=\frac{1}{\beta }ln\frac{∆P_{0}}{∆P}=L_{p}\;\frac{t}{l}\;$$

where the pressures in the feed compartment and permeate compartment are ‘*P_feed_*’ [bar] and ‘*P_perm_*’ [bar], respectively. ‘*L_p_*’ [m^2^s^−1^] is the permeability of a membrane of thickness ‘*l’* [m] and ‘*t’* [s] is the time. ‘*β*’ [m^−1^] is the characteristic of the geometry of the cell calculated by the Equation (5) where, ‘*A’* [m^2^] is the membrane area and ‘*V_feed_*’ and ‘*V_perm_*’ are the volumes of the feed compartment and permeate compartment, respectively.

(5) $$\beta =A\left(\frac{1}{V_{feed}}+\frac{1}{V_{perm}}\right)\;$$

The permeability of the gas was calculated from the slope obtained by plotting $\left(\frac{1}{\beta }ln\frac{∆P_{0}}{∆P}\right)$ versus $\left(\frac{t}{l}\right)$. Subsequently, the ideal selectivity, ‘*α*’, was calculated by dividing the permeability of the more permeable gas with the permeability of the less permeable gas.

## 3. Results and Discussion

### 3.1. Formulation of Water-in-Oil Emulsions by Membrane Emulsification

#### 3.1.1. Sorption Studies for Screening of Commercials Oils as Continuous Phase

Regular food-grade oils were chosen as the oil phase for the bulk of the emulsions. The sorption results obtained at 30 °C are reported in Table 2. The various oils presented similar sorption coefficients for CO_2_. Considering the cost, either sunflower oil or corn oil could have been chosen as the continuous phase. However, since the CO_2_ sorption of corn oil was slightly higher, it was chosen as the continuous phase. 

#### 3.1.2. Screening of Surfactants for Dispersed Phase

**Figure 6:**
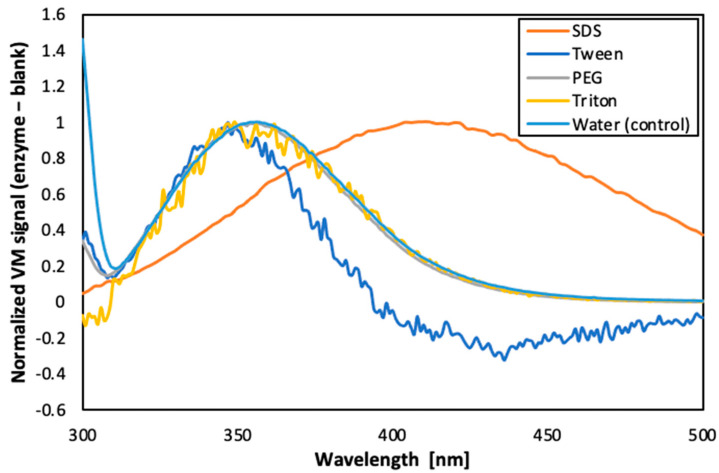
Fluorescence emission spectra of carbonic anhydrase in presence of different surfactants.

A fluorescence analysis of the enzyme in the presence of different surfactants was performed (as shown in the Figure 6). The results suggest that PEG 300 was the surfactant that best preserved the conformation of the enzyme, since only slight band shifts were observed (curve almost overlapped with the control sample). On the other hand, Tween and SDS caused drastic changes in the conformation of the enzyme, from the point of view of exposure of the aromatic residues of tryptophan to the solvent. Additionally, the concentration of PEG 300 to be used was optimised as 5% (*v*/*v*). Therefore, this condition was selected for the next phase of the study. 

Furthermore, as reported in literature, PEG is known to stabilise enzymes by forming hydrogen bonds, which could displace water and prevent protein aggregation [32]. Furthermore, PEG has also been reported to reduce the viscosity ratio between the dispersed and the continuous phase (by enhancing the dispersed phase viscosity) which contributes to shrinkage of the emulsion droplets [33,34]. 

#### 3.1.3. Optimisation of the Operating Conditions to Formulate Water-in-Oil Emulsions

**Figure 7:**
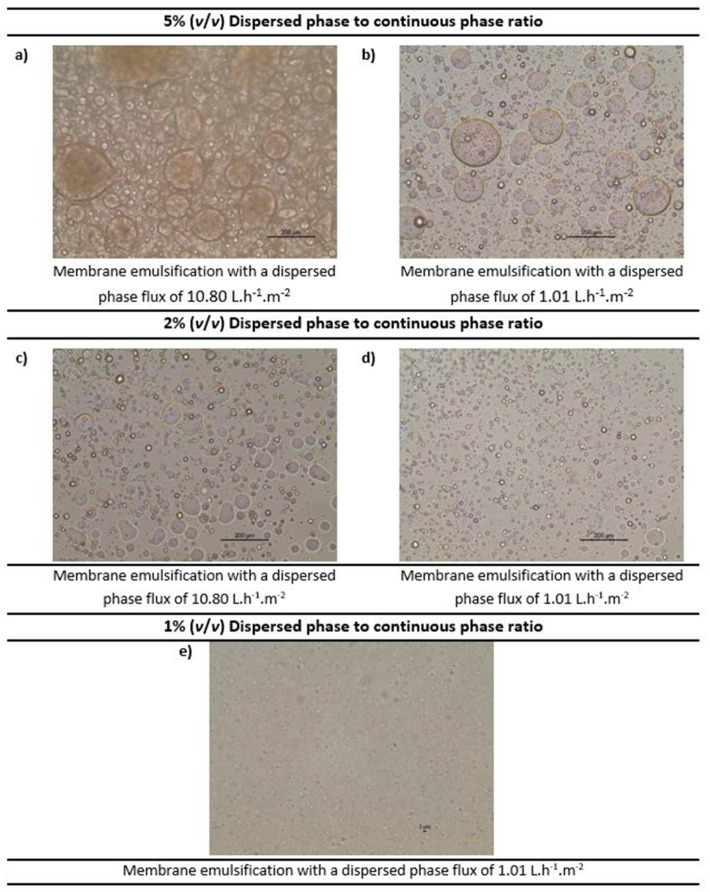
Optimisation of the water-in-oil emulsions (at constant crossflow velocity of the continuous phase) by optical microscopy. Note: The scale shown in the images 6 (**a**–**d**) is 200 µm and in 6 (**e**) is 3 µm.

Membrane emulsification was performed to formulate water-in-oil emulsions both without and with the functional agent (the carbonic anhydrase enzyme) using the methodology detailed in Section 2.4. Various emulsions were prepared (both without and with carbonic anhydrase) with volumetric ratio of dispersed phase to continuous phase ranging from 1%, to 2% and 5%. The dispersed phase flux was also varied (10.80 L.h^−1^.m^−2^ and 1.01 L.h^−1^.m^−2^) in order to understand its impact. The continuous phase cross flow velocity was kept constant at 0.15 m.s^−1^, which corresponds to wall shear stress of 11.9 Pa. Moreover, 2% (*w*/*w*) of Tween 80 was optimised as the best surfactant concentration in the continuous phase as the other surfactants and concentrations tested were either not able to emulsify completely, or the resultant emulsions had excess of surfactant. The different emulsions obtained were further characterised by optical microscopy (refer Figure 7).

As observed in Figure 7, as we reduce the volume ratio, the emulsion droplets become smaller. For 5% concentration (Figure 7a,b) and 2% concentration (Figure 7c,d), the droplets are in the micro-meter range, while for the 1% concentration (Figure 7e) the droplets are so small that they are barely visible. Moreover, it was observed that the lower dispersed phase flux (based on the dispersed phase flowrate) promotes the production of more uniform emulsions (see Figure 7d). These results were consistent with the literature [23,35]. Hence, emulsions formed by a 1% volume ratio of dispersed phase to continuous phase and dispersed phase flux of 1.01 L.h^−1^.m^−2^ were selected for further investigation.

Based on visual observation, a stable emulsion was formed. However, as the bulk of the emulsions formulated in this study were made using corn oil (an oil with yellowish colour), inconclusive results were obtained when characterised by DLS. 

### 3.2. Synthesis and Characterisation of Water-in-Oil Emulsions by Membrane Emulsification

#### 3.2.1. Interfacial Tension Studies of the Two Phases

**Figure 8:**
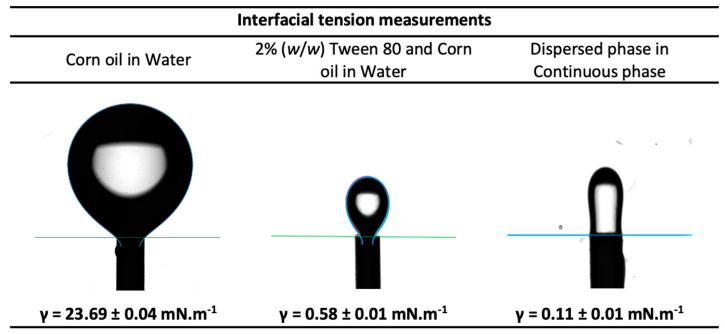
Interfacial tension measurements of the continuous phase and dispersed phase.

The interfacial tension of the dispersed phase and of the continuous phase, measured by the pendant drop method, are shown in Figure 8. According to the literature, the interfacial tension of conventional hydrophobic oils with water are in the range of 22–26 mN.m^−1^ [36]. In the present study, the interfacial tension of corn oil with water was determined to be 23.69 ± 0.04 mN.m^−1^, which is on par with literature. On the addition of surfactant (2% (*w*/*w*) Tween 80) to the corn oil (the continuous phase), the interfacial tension reduced significantly, showing an interfacial tension value of 0.58 ± 0.01 mN.m^−1^. The reduction of the interfacial tension by emulsifiers can be attributed to the quick adsorption of the emulsifier onto the interface of immiscible liquids to promote their interaction [19,20]. There was a further reduction in the interfacial tension value to 0.11 ± 0.01 mN.m^−1^ between the dispersed phase and the continuous phase where the dispersed phase consisted of water and 5% PEG 300. This drastic reduction in the interfacial tension in presence of Tween 80 surfactant to values close to zero facilitates the formation of droplets of the dispersed phase in the continuous phase.

#### 3.2.2. Contact Angle Measurements Associated with the Emulsification Process

**Figure 9:**
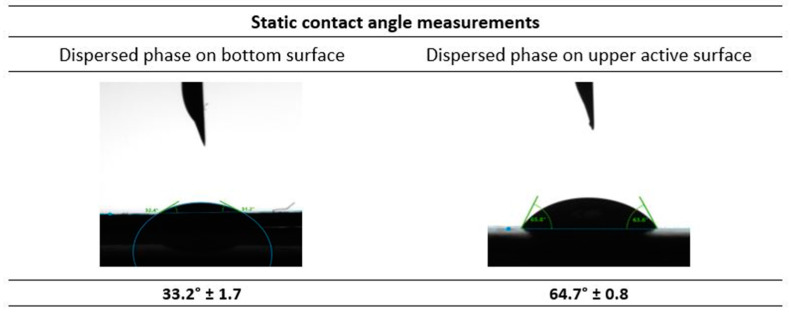
Static contact angle measurements of the dispersed phase with the membrane selected for the membrane emulsification studies.

The contact angle of the top (active) and bottom surfaces of the UP150, Polyethersulfone (PES) membrane were measured with respect to the dispersed phase (Water + 5% PEG 300 + Carbonic anhydrase). The results depicted in Figure 9 show that the contact angle of the dispersed phase with the bottom surface of the membrane is 33.2° ± 1.7. This implies that the dispersed phase easily wets the surface, contributing to lower the pressure required for the membrane emulsification process. Furthermore, the contact angle for the dispersed phase with the opposite active upper side of the membrane was found to be 64.7° ± 0.8. This value shows that the upper side of the membrane is relatively more hydrophobic than the bottom side, which facilitates the detachment of the emulsion droplets formed, to the continuous phase. So, in the presence of a cross-flow shear from the continuous phase, the formation of emulsion droplets is favoured. This is in favour of the formation of emulsions, as reported previously in [35]. As the dispersed phase passes through the membrane pores, the continuous phase is able to detach from the membrane surface to form emulsions.

### 3.3. Gas Transport Studies of CO_2_ and CH_4_ through the Synthesised Membranes

The effect of the inclusion of carbonic anhydrase in the emulsion was studied by measuring the pure gas permeability of CO_2_ and CH_4_. The single gas permeabilities were measured with a 69.9 kPa transmembrane pressure at 30 °C. The ideal gas permeability was calculated as a ratio of the two permeabilities. Firstly, the permeabilities of the SLM, formed by the hydrophobic PVDF support and corn oil, were tested and then compared with the permeabilities of the SLMs formed by introduction of the emulsions within the porous structure of a similar membrane support. It was observed that for the SLM and the emulsion based SLM without carbonic anhydrase, the permeabilities were almost similar (see Table 3). This was expected as the emulsion-based SLM without carbonic anhydrase has no components to facilitate the transport of CO_2_. On the contrary, upon inclusion of carbonic anhydrase in the emulsions, there was a drastic improvement in the CO_2_/CH_4_ selectivity. This can be attributed to the facilitated transport of CO_2_ by the carbonic anhydrase. It was noted that along with the facilitated CO_2_ transport, CH_4_ transport was further reduced, which led to a higher selectivity of emulsion-based SLMs in comparison to SLMs. We know that CH_4_ is a non-polar molecule. Hence, upon the introduction of water (polar solvent) as dispersed phase, the permeability of CH_4_ slightly decreased. Moreover, London dispersion forces (dominant in nonpolar molecules) are much weaker than dipole–dipole interactions (dominant in polar solvents). Therefore, the propensity of non-polar molecules to interact with polar solvents is minimum. This is because of the energy released due to formation of dispersion forces between such molecules is not enough to break strong dipole–dipole interactions between polar molecules. Hence, CH_4_ transport is further inhibited in presence of water in the emulsion droplet. For the emulsions with carbonic anhydrase, the dispersed phase is set at a pH of 11 (for better enzymatic activity). This basic pH further reduces the CH_4_ solubility and hence its transport, as reported in the literature [37].

**Figure 10:**
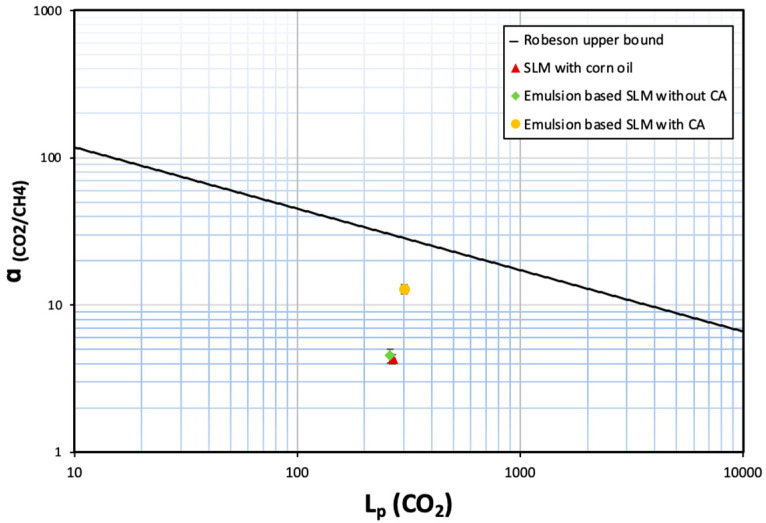
Comparative analysis of SLMs with conventional Robeson upper-bound plot.

It is interesting to note that even such a small amount of carbonic anhydrase (<4 ppm in the emulsion retained by the supported liquid membrane) can enhance the selectivity by ~3×. The novelty and relevance of this work lies in establishing a proof of concept that demonstrates how the incorporation of carbonic anhydrase in an emulsion immobilised in a porous membrane substrate enhances the selective recovery of CO_2_ from CH_4_. When the CO_2_/CH_4_ selectivity of these membranes is plotted as a function of CO_2_ permeability, the membranes are not above the Robeson upper bound plot [38]. Still, there is a clear indication that incorporation of carbonic anhydrase in nano/micro droplets clearly improves the performance of the membrane for biogas separation (see Figure 10).

## 4. Conclusions

A detailed experimental methodology was developed in this study to explore the CO_2_/CH_4_ separation capabilities of a bio-based emulsion system impregnated onto a support (porous membrane). Carbonic anhydrase was chosen as the active functional agent to enhance the CO_2_ transport property of the membrane. An energy efficient and mild direct membrane emulsification technique was effective in formulating the enzyme-based emulsions. Corn oil with 2% (*w*/*w*) Tween 80 was used as the continuous phase of the emulsion. The dispersed phase was 0.5 g.L^−1^ carbonic anhydrase enzyme with 5% PEG 300 (*w*/*w*) in aqueous potassium carbonate solution. Nadir^®^ UP150 P flat-sheet polymeric membranes (Microdyn-Nadir GmbH, Wiesbaden, Germany) were used to produce emulsions. 

The resulting emulsions were subsequently impregnated onto a microporous hydrophobic PVDF membrane (nominal pore size 0.22 µm) to prepare emulsion-based supported liquid membranes. For comparative studies, the supported liquid membrane (SLM) was prepared by impregnating corn oil onto the membrane and emulsion-based supported liquid membranes were also prepared both without and with the carbonic anhydrase enzyme. It was observed that the permeability of CO_2_ increased by ~15% and that of CH_4_ decreased by ~60% through the emulsion-based SLM containing carbonic anhydrase when compared to the SLM and emulsion-based SLM without carbonic anhydrase. Subsequently, the selectivity of CO_2_ increased in the presence of carbonic anhydrase. It must be stressed that the current study aims to establish a new proof-of-concept through the development of a supported liquid membrane with immobilised emulsions, loaded with a low concentration of functional agent (carbonic anhydrase, in this case). Moreover, it can be safely presumed that loading higher concentration of carbonic anhydrase in the emulsion droplets might contribute in further facilitated transport of CO_2_, thereby taking the performance of the membranes further close or beyond the Robeson upper bound plot. Furthermore, only the ideal selectivity of gases was evaluated in this work. However, it will be relevant to test the mixed gas selectivity. This sets the premise for further investigation on exploiting such emulsion-based supported liquid membranes.

## Figures and Tables

**Table 1:** Details of commercial membranes used in this study.

Membrane Used	Membrane Specifications	Objective
UP 150 P Nadir^®^	Polyethersulfone (PES), 150 kDa (MWCO), 26 nm nominal pore size	Formulation of water-in-oil emulsions by membrane emulsification
Durapore^®^ Membrane Filter	Hydrophobic Polyvinylidene fluoride (PVDF), 0.22 µm pore size	Support for the supported liquid membranes

**Table 2:** Sorption coefficients of oils at 30 °C.

Oils	Sorption Coefficient (cm^3^(*STP*)/(cm^3^.atm)
Corn Oil	30.31 ± 0.23
Olive Oil	29.85 ± 0.05
Sunflower Oil	29.94 ± 0.05

**Table 3:** Permeabilities of CO_2_ and CH_4_ and the selectivity of different membranes.

Membrane		L_p_ (CO_2_) (Barrer)	L_p_ (CH_4_) (Barrer)	Selectivity (α _(CO_2_/CH_4_)_)
Support	Filler			
Hydrophobic PVDF	Corn Oil	267.40 ± 3.60	62.00 ± 3.70	4.31 ± 0.20
	Emulsion without carbonic anhydrase	259.90 ± 3.30	57.30 ± 5.50	4.54 ± 0.40
	Emulsion with carbonic anhydrase	301.85 ± 7.45	23.55 ± 0.65	12.82 ± 0.10

## Data Availability

The data presented in this study are available on request from the corresponding author.
